# The Mediating Role of Mental Toughness in the Relationship Between Burnout and Perceived Stress Among Hungarian Coaches

**DOI:** 10.3390/sports13060174

**Published:** 2025-05-31

**Authors:** Eszter Bíró, László Balogh

**Affiliations:** 1 Institute of Sport Sciences, University of Debrecen, 4032 Debrecen, Hungary; balogh.laszlo@sport.unideb.hu; 2 Faculty of Health Sciences, Doctoral School of Health Sciences, University of Pécs, 7624 Pécs, Hungary

**Keywords:** burnout, coach well-being, stress, structural equation modeling, sport psychology, mental toughness

## Abstract

This study aimed to explore the relationship between perceived stress, burnout, and mental toughness, and investigate how mental toughness mediates the relationship between perceived stress and burnout among Hungarian coaches. Three hundred and thirty-three active coaches completed anonymous self-report questionnaires assessing burnout (CBQ), mental toughness (SMTQ), and perceived stress (PSS). In the international comparison, Hungarian coaches indicated significantly higher burnout levels than those investigated in previous international research. PLS-SEM results showed that coaches with higher stress levels are less likely to experience burnout. Furthermore, mental toughness serves as a buffer between stress and burnout; however, the buffering effects of its various subfactors on different dimensions of coaching burnout vary. Consequently, further research is necessary to fully understand the mitigating effects of mental toughness, particularly by analyzing coping strategies, motivation, and success. Our research unequivocally indicates that the integration of targeted stress management training into coaching education is crucial for effectively addressing the emotional challenges inherent in the profession. By incorporating workshops that focus on self-awareness and emotion regulation strategies, coaching programs can substantially reduce the risk of burnout and promote the well-being of coaches. These findings underscore the vital importance of embedding practical stress management interventions within coach development initiatives to cultivate more resilient and effective coaching professionals.

## 1. Introduction

The role of a coach is complex and comes with significant expectations at various levels of sport. In this dynamic and often contradictory environment, coaches must perform multiple roles [1]. They undertake a wide range of responsibilities, including educating, teaching, imparting knowledge, developing skills, and monitoring both learning and performance. Additionally, they must respond to and provide feedback for their athletes. Coaches also focus on the development and personality shaping of their athletes; however, their primary objective is to help athletes reach their maximum potential, as their work is evaluated against the standards of the sport [2]. The coaching profession requires preparation in both professional knowledge and pedagogical strategies to achieve success. For a long-term and successful coaching career, professional versatility is essential. In addition to mastering conditional knowledge and technical and tactical expertise, coaches must clearly formulate sports-related goals. Ethical considerations also play a significant role in this field, as coaches bear responsibility for the lives and health of the athletes entrusted to them [3,4]. Coaching is characterized by several traits: it is complex, goal-oriented, evaluative, continuous, thought-provoking, reliant on environmental factors, intricate, requiring adaptation to available resources, strategic, and consequential [5].

As Hans Selye, regarded as the father of stress research, famously stated, “Stress is the essence of life” [6]. In our constantly changing world, the environment subjects us to a perpetual state of stress [7]. Stress is response-based, conceptualizing it chiefly in terms of the body’s physiological reaction to any demand placed upon it [8] (p. 25). Due to the financial pressures associated with sports, expectations for both athletes and coaches are continually increasing, leading to heightened performance pressure. Consequently, their work is conducted under ongoing stress. Excessive and chronic stress can lead to physical changes in the body, such as damage to various immune system functions and increased cortisol levels [9]. It may also result in mental and behavioral problems, including depression and a general sense of malaise [10]. Furthermore, performance may decline, eventually leading to burnout [11,12]. A crucial long-term consequence of stress that directly affects daily life is burnout [13].

In sports, coaching burnout is a significant issue that adversely affects both athletes and teams [14]. Burnout results in a decline in coaches’ enthusiasm, performance, motivation, training participation, and communication skills, ultimately undermining their credibility [15,16,17]. This condition often coincides with an internal crisis and feelings of dissatisfaction, leading coaches to struggle to find meaning in their work and to question their career advancement opportunities [18]. Consequently, this can result in a loss of self-confidence, which undermines the relationship between coaches and athletes [16,17].

The phenomenon of burnout in the sports environment was first studied among coaches. Initially, researchers focused on demographic and sport-specific variables. Their findings revealed that female collegiate coaches experienced higher levels of emotional exhaustion than their male counterparts, attributing this to the greater pressure they face in competing against men for their positions and dealing with increased financial uncertainty [19]. In the following decade, numerous analyses of collegiate coaches further supported these findings [20,21,22,23]. Later research in Europe examined gender differences in burnout, but found no significant disparities between male and female coaches [24,25,26,27]. However, recent studies indicate that women reported higher average burnout scores than men [28,29]. Overall, research on gender differences in burnout shows little significant disparity. Most studies indicate that emotional exhaustion and low job satisfaction are more frequently reported among women, while men more commonly experience depersonalization and a decline in personal performance [30,31].

Mental toughness is defined as having a natural or developed psychological edge that enables performers to cope better than their opponents with the demands and pressures of competing at the highest level in sport. Specifically, these performers are more consistent than and superior to their opponents in remaining determined, focused, confident, and in control under pressure [32] (p. 83). Mental toughness refers to a resilient mindset characterized by confidence, control, commitment, and a willingness to challenge oneself [33].

Mental toughness shares similarities with other psychological concepts, such as resilience, perseverance, self-efficacy, motivation, and emotional regulation, all of which relate to how individuals respond to challenging circumstances [34]. However, it also possesses a distinct, proactive quality that enables individuals to perform well even in positively charged environments [35].

Several pieces of empirical evidence support the protective role of mental toughness in the burnout process. Generally, individuals with higher levels of mental toughness are better equipped to manage the demands associated with sports. Such individuals tend to perceive stressors as manageable challenges rather than threats, which facilitates adaptive coping strategies and perseverance [36,37]. 

Researchers hold differing opinions regarding the components of mental toughness. Some support a unidimensional model [38,39], while others advocate for a multidimensional approach [33,37]. Currently, the most widely accepted model among professionals is the four-dimensional framework proposed by Clough et al. [33], which comprises the following elements: control, commitment, challenge, and confidence.

Control refers to an individual’s belief in their ability to influence their own life and emotional state amidst various circumstances. Commitment reflects perseverance toward goals despite difficulties, indicating a tendency to prioritize engagement in experiences over withdrawal. Challenge pertains to the belief that change, rather than stability, is the norm in life, and that preparing for change serves as a motivating factor for growth rather than a threat to security. Furthermore, challenge involves viewing potential threats as opportunities for personal development. Lastly, confidence signifies feeling valuable and competent in addressing general and interpersonal problems as they arise. Maintaining confidence entails preserving self-esteem and belief in one’s ability to progress socially, even in the face of setbacks [33].

Additionally, individuals with higher levels of mental resilience possess exceptional psychological skills, a clear understanding of their priorities, and exhibit elevated self-confidence and unwavering beliefs [40]. When faced with setbacks or losses during training or competition, they can perceive these negative events as opportunities for problem identification and resolution, thereby mitigating the adverse effects of adversity and maintaining their focus. This constructive emotional cognition enables athletes to inhibit feelings of fear, suppress negative thoughts, foster positive emotions, and enhance their adaptive capacity. Consequently, adolescent athletes are better equipped to regulate their emotional responses, demonstrate superior emergency coping skills, and, as a result, exhibit increased levels of engagement in their athletic pursuits [41].

Overall, stress is a key antecedent of burnout. However, individual differences influence how stress manifests as burnout, with mental toughness identified as a crucial psychological resource that can mitigate adverse outcomes. Mental toughness is a comprehensive term encompassing positive psychological resources that are significant across various contexts [33]. It serves not only as an effective coping mechanism in response to stressors, such as reinterpreting stressful situations as opportunities for self-development, but also enables individuals to proactively pursue personal growth due to their high levels of confidence in their abilities [34]. It mitigates risks, minimizes negative chain reactions, and fosters self-esteem and self-efficacy [42]. 

An analysis of gender differences among Swiss athletes revealed that women reported higher levels of stress and depressive symptoms, while men exhibited greater mental toughness. Higher stress levels in young athletes were associated with lower mental toughness, which correlated with reduced burnout, anxiety, and depressive symptoms, highlighting mental toughness as a protective factor against burnout [43,44]. This finding aligns with research on police officers and firefighters [45], high school and university students [44,46], vocational students [47], esports athletes [48], young athletes [49,50], and Chinese weightlifters [51], all indicating that elevated mental toughness is linked to lower burnout levels. Notably, while burnout symptoms were minimal at low perceived stress levels, individuals with low mental toughness reported increased symptoms during high-stress periods [43]. Additionally, higher stress levels correlate with more mental health issues, and those with lower mental toughness experience greater perceived stress and reduced quality of life, with work duration also significantly affecting these outcomes [46,47]. 

In recent years, numerous studies have investigated the mediating effects of emotional labor [52], resilience, stress, defense mechanisms, and recovery on burnout [53]. However, most research has focused on athletes [52,53,54,55]. Despite this, Gerber et al. explored the relationship between mental toughness, stress, and burnout among adolescents; to our knowledge, there is currently no research examining the relationship between perceived stress, mental toughness, and burnout among coaches [43,47].

This study aims to fill a significant gap in the existing literature by investigating the mediating role of mental toughness in the relationship between perceived stress and burnout among coaches. The increasing expectations, performance pressures, and intensity of match and competition schedules underscore the necessity of understanding the factors contributing to burnout and providing protection against it.

Consequently, this paper has three main objectives: (a) to explore differences in burnout levels between Hungarian coaches and those reported in international research; (b) to investigate the relationship between perceived stress, burnout, and mental toughness; and (c) to explore the mental toughness factors (Confidence, Constancy, Control) as mediators of the relationship between perceived stress and burnout among Hungarian coaches. (H1) We do not expect burnout levels among Hungarian coaches to differ from international data. Furthermore, we hypothesize that (H2) stress may be positively linked to burnout. Additionally, we suggest that (H3) mental toughness could buffer the relationship between perceived stress and burnout among Hungarian coaches

## 2. Materials and Methods

### 2.1. Participants

Previous research indicates that a sample size ranging from 100 to 200 is generally considered an appropriate starting point for conducting path modeling [48]. A total of three hundred and thirty-three active coaches (108 females and 225 males) aged 19 to 78 years (M = 43.08 years, SD = 12.95) participated in the study. As the survey was conducted online, it could not be considered complete without full responses; consequently, there are no missing values in the dataset. The coaches represented 36 different sports, and more than 50% of the sample had over 10 years of coaching experience (see Table 1). Participants were volunteers and did not receive any compensation for their participation.

### 2.2. Measures

#### 2.2.1. Coach Burnout

The adaptation of the Coach Burnout Questionnaire, developed by Harris and Ostrow [56], into Hungarian is attributed to Bíró et al. [57]. This questionnaire subjectively analyzes three main areas: Emotional and Physical Exhaustion (EPE), Reduced Sense of Accomplishment (RSA), and Sport Devaluation (SD). It consists of 15 statements, on which respondents indicate their feelings on a 5-point Likert scale (1 = almost never, 2 = rarely, 3 = sometimes, 4 = often, and 5 = almost always). The subscale for Emotional and Physical Exhaustion comprises eight items, the dimension of Reduced Sense of Accomplishment consists of four items, and the dimension related to Sport Devaluation includes three items. For the negatively worded items (2–13, 15), reverse scoring was applied. Higher scores indicate a greater level of burnout. Previous research has consistently revealed that the CBQ had satisfactory validity and reliability among Hungarian coaches [57]. The Cronbach’s alpha for the total CBQ was 0.69 in this study. The Composite Reliability of the Emotional and Physical Exhaustion, Reduced Sense of Accomplishment, and Sport Devaluation subscales was 0.907, 0.788, and 0.765, respectively.

#### 2.2.2. Mental Toughness

The Hungarian version of the Sport Mental Toughness Questionnaire [58], developed by Sheard et al. in 2009 [59], was used to measure mental toughness. This self-report questionnaire contains 14 items on a four-point Likert scale, ranging from “not at all true” to “very true”. The Mental Toughness Questionnaire comprises three subscales: Confidence, Constancy, and Control. For negatively worded items (2, 4, 7–10), reverse scoring was applied during the evaluation. The Hungarian version of the SMTQ demonstrated adequate internal consistency and validity in Hungarian athletes [58]. The Cronbach’s alpha of the total scale was 0.64 in this study. The Composite Reliability of the Confidence, Constancy, and Control subscales was 0.856, 0.792, and 0.776, respectively.

#### 2.2.3. Perceived Stress

The questionnaire developed by Cohen et al. [60], known as the Perceived Stress Scale, is one of the most widely used tools for assessing stress levels. The Hungarian adaptation of this scale is credited to Stauder, who validated the scale among Hungarian adults [61]. The questionnaire consists of inquiries regarding fundamental thoughts and feelings, aimed at evaluating the stress perception of the individuals being studied. The questions pertain to a one-month period, ensuring that daily events do not influence the substantive responses. A level of education beyond elementary school is not required to complete the questionnaire, as the questions are straightforward and easily comprehensible. It consists of 14 items, which respondents score on a five-point Likert scale (0 = never, 1 = almost never, 2 = sometimes, 3 = fairly often, and 4 = very often). For certain items, higher scores indicate a greater frequency of stress situations and a more effective coping mechanism. After summing the scores for the responses, a global indicator of perceived stress is obtained. It is important to note that scoring is reversed for items 4, 7, 9, 10, and 13. The PSS has demonstrated reliability and effective operationalization, with its construct and predictive validity appropriately substantiated [61]. The Cronbach’s alpha for the total PSS was 0.77. The Composite Reliability of the Perceived Distress, Perceived Coping, and Perceived Anxiety subscales was 0.882, 0.879, and 0.790, respectively, in this study.

### 2.3. Procedure

The research was part of the Thematic Excellence Program—Health Subprogram (TKP 2021-EGA-20), approved by the Ethics Committee of the University of Debrecen (Protocol Number: 6088-2022), and adhered to the principles of the Declaration of Helsinki. In this study, a purposeful sampling strategy was applied, with email addresses compiled from the official websites of the sports federations. Our questionnaire was distributed as a web survey to 903 active email addresses and social media platforms (e.g., Facebook, Viber, iMessage). Of these, 333 participants completed a usable questionnaire, resulting in a response rate of 36.9%. Data collection occurred from 12 November 2024 to 22 January 2025. All individuals participating in this study had to be active coaches in the current sport season. Participation was voluntary, and written informed consent was obtained before data collection. Participants were not compensated, but we express our gratitude for their contributions.

### 2.4. Data Analysis

**Figure 1:**
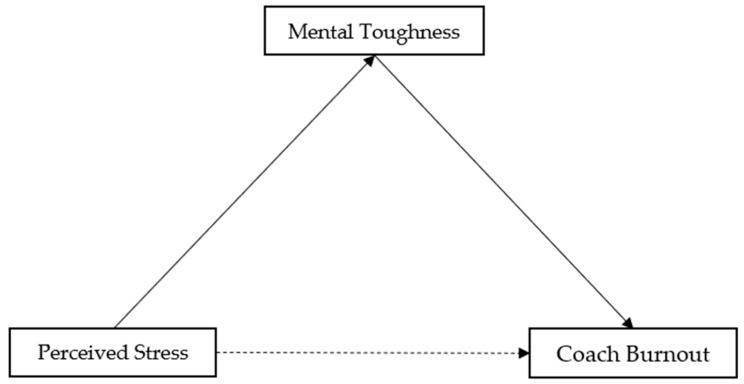
The illustration of the analyzed paths.

Data analysis was performed using IBM SPSS Statistics (Version 20) and Smart PLS 4 software [54]. Structural Equation Modeling (SEM) was conducted with Smart PLS 4 to investigate the mediating effect of mental toughness on the relationship between perceived stress and burnout (see Figure 1). Cronbach’s alpha tends to provide a conservative measurement in PLS-SEM; prior research has suggested using Composite Reliability as an alternative [62,63]. Composite Reliability values should be 0.70 or higher [62]. The results met this criterion, with construct values ranging from 0.64 to 0.77 for α and between 0.77 and 0.88 for Composite Reliability. The validity and collinearity of the questionnaires were assessed through outer loadings (≥0.05), the Fornell–Larcker criterion, cross-loadings, the Heterotrait–Monotrait ratio (HTMT), and the Variance Inflation Factor (VIF). The Standardized Root Mean Square Residual (SRMR) was calculated to demonstrate the model fit. The outer loadings ranged from 0.50 (PSS12) to 0.875 (SMTQ7), and the VIF values ranged from 1.02 (PSS12) to 2.96 (CBQ4). An SRMR value between 0.10 and 0.08 is considered a good fit [64]. Our results showed an SRMR value of 0.076. These indices provided a comprehensive assessment of how well the proposed model represented the data, with acceptable values indicating a good fit. We also analyzed the direct, indirect, and total effects of perceived stress, mental toughness, and burnout. A bootstrapping procedure was performed to generate t-statistics to assess the significance of the structural paths, utilizing the recommended number of 5000 bootstrap repetitions [63,65]. 

## 3. Results

### 3.1. Demographic Characteristics

Descriptive statistics regarding age, gender, coaching levels, and coaching experience are summarized in Table 1, which displays the means and standard deviations for continuous variables, as well as frequencies and percentages for categorical variables. Among the 333 coaches, the average age was 43.08 ± 12.95 years, with 108 (32%) identifying as female. The years of coaching experience among participants ranged from 1 to over 51 years. Coaching experience was classified according to Verger and Lee [66]. Nearly half of the coaches were employed in the First Class and/or National Team, while the remaining coaches were engaged at lower levels (see Table 1).

The coaches represented a total of 34 sports, including both individual and team disciplines, with the distribution illustrated in the following table (Table 2).

### 3.2. Descriptive Statistics

Descriptive statistics were calculated to investigate the study variables. The skewness and kurtosis measures were found to be within acceptable ranges, from −0.7 to 1.24 for all variables (see Table 3).

### 3.3. Comparison of Burnout Values with International Data

We compared the averages of the burnout subfactors with certain international burnout data (See Table 4). A one-sample *t*-test was employed to examine the differences between the means obtained in our research and those reported in various international studies. We noted with concern that, for Emotional and Physical Exhaustion, Reduced Sense of Accomplishment, and Sport Devaluation, the average values were nearly double (*p* < 0.001) those found in international research.

### 3.4. Mediation Analysis

Overall, perceived stress significantly negatively affects coach burnout and mental toughness. This means that Hungarian coaches with higher perceived stress are less likely to experience burnout. Furthermore, surprisingly, mental toughness has a significant positive impact on coach burnout (see Table 5). In our model, mental toughness does not buffer the relationship between overall stress and overall burnout. Numerous research findings and theoretical frameworks propose potential explanations for the paradoxical relationship between mental toughness and burnout. Individuals exhibiting high levels of mental toughness often demonstrate considerable resilience, self-confidence, and perseverance [33]. Although these characteristics can be advantageous, they may also predispose individuals to perfectionistic tendencies and overcommitment, which can lead to heightened stress and burnout when expectations are not met or when workloads become excessively burdensome [71,72].

**Figure 2:**
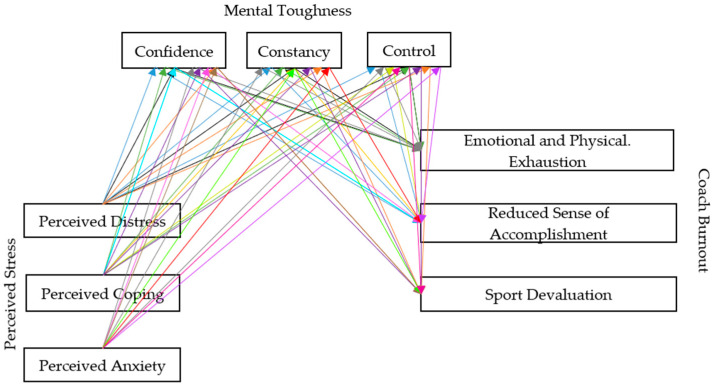
The illustration of the analyzed paths for all subscales.

Furthermore, the significantly negative relationship identified between stress and mental toughness in the present study can be theoretically attributed to the principles outlined by transactional stress theorists [73,74]. Stress is defined as a state of imbalance between internal or external demands and the personal or social resources available to manage a potential stressor. Individuals with high mental resilience are characterized by a pronounced tendency to perceive their environment as controllable, view themselves as capable and influential, remain committed even in adverse circumstances, and interpret problems as natural challenges that facilitate personal growth [33]. To better understand these contradictory results compared to previous research, mediation analysis was conducted for all subscales (See Figure 2).

The results revealed that all PSS factors—Perceived Distress (β = −0.369, *p* < 0.001), Perceived Coping (β = −0.139, *p* < 0.05), and Perceived Anxiety (β = −0.328, *p* < 0.001)—were negatively associated with Emotional and Physical Exhaustion. Among the three observed stress factors, Perceived Distress had the most significant direct impact on Emotional and Physical Exhaustion, while the greatest indirect effect of the Mental Toughness subfactors was detected on Perceived Coping. The Mental Toughness factors had an inverse mediating effect on all burnout subfactors. However, Mental Toughness subfactors did not moderate the relationship between Perceived Stress, Perceived Anxiety, and Emotional and Physical Exhaustion, but did moderate the relationship between Perceived Coping and Emotional Exhaustion (see Table 6).

Higher Perceived Distress and Perceived Anxiety correlate with lower Emotional and Physical Exhaustion. While Perceived Distress pertains to an individual’s subjective experience of stress or discomfort, it does not necessarily correspond to physical or emotional exhaustion. Certain individuals may perceive elevated levels of distress, yet effectively regulate or compartmentalize their emotional responses through appropriate coping strategies, thereby preventing exhaustion [74]. This implies that Perceived Distress may, at times, represent an increased awareness or engagement with stressors rather than indicators of burnout. The Constancy subfactor reflects determination, personal responsibility, an unwavering mindset, and the ability to maintain focus [59]. This entirely substantiates why an individual may not experience exhaustion when this factor is present in the relationship between stress and burnout.

Perceived Distress (β = −0.107, *p* = 0.155), Perceived Coping (β = −0.055, *p* = 0.432), and Perceived Anxiety (β = −0.111, *p* = 0.062) have no statistically significant direct effect on Reduced Sense of Accomplishment. However, regarding the indirect effects, a statistically significant impact of Mental Toughness factors was found between Perceived Distress (β = −0.267, *p* < 0.001), Perceived Coping (β = −0.317, *p* < 0.001), Perceived Anxiety, and Reduced Sense of Accomplishment (β = −0.240, *p* < 0.001). Despite this, no statistically significant indirect effect of the Constancy subfactor was detected between Perceived Distress and Reduced Sense of Accomplishment (see Table 7). Consequently, stress factors have no impact on a Reduced Sense of Accomplishment. Furthermore, if confidence influences the relationship between Perceived Distress, Perceived Anxiety, and a Reduced Sense of Accomplishment, individuals are less likely to experience a decrease in their performance. If the subfactors of Mental Toughness (Confidence, Constancy, Control) are considered in the relationship between stress and burnout, the individual will be less likely to feel a decrease in their sporting performance.

None of the perceived stress factors—Perceived Distress (β = −0.052, *p* = 0.449), Perceived Coping (β = 0.102, *p* = 0.121), or Perceived Anxiety (β = −0.104, *p* = 0.110)—have a statistically significant direct effect on Sport Devaluation. However, Mental Toughness negatively impacts all three perceived stress subfactors. Despite this, no statistically significant moderating effect of the Control subfactor was detected between perceived stress subfactors and Sport Devaluation (see Table 8). Consequently, it can be stated that Confidence and Constancy protect individuals from perceived stress, while Control has no impact on perceived stress.

## 4. Discussion

The world of sports, characterized by a series of matches, competitions, and an unwavering pursuit of excellence, renders coaches more vulnerable to chronic stress, a significant precursor to burnout. Investigating the mediating effects of mental toughness may enhance our understanding of this relationship. Therefore, the purposes of the study were (a) to explore differences in burnout levels between Hungarian coaches and those reported in international research; (b) to investigate the relationship between perceived stress, burnout, and mental toughness; and (c) to explore mental toughness factors (Confidence, Constancy, Control) as mediators of the relationship between perceived stress and burnout among Hungarian coaches.

### 4.1. First Hypothesis

Contrary to our first hypothesis, our research revealed significantly higher burnout levels across all subfactors—Emotional and Physical Exhaustion (*p* < 0.001), Reduced Sense of Accomplishment (*p* < 0.001), and Sport Devaluation (*p* < 0.001)—among Hungarian coaches compared to previous international results. Notably, this study identified exceptionally high burnout scores, nearly double for all three subfactors. According to data from 2021, 7% of Hungarians over the age of 15 experience depression. Moreover, Hungary has had one of the highest suicide mortality rates in the world for decades [75]. A recent study shows that Hungarians are more dissatisfied with their lives than the general population of the European Union [76]. Overall, we believe that the underlying reasons for this may stem from cultural and moral differences, as supported by numerous studies. The findings of these studies, along with our own research, substantiate the existence of cultural and moral disparities between Hungarians and people from other nations.

These results underscore the urgent need for further investigation into the underlying factors contributing to the significant level of coach burnout. Additionally, we aim to develop management strategies for professional associations and sports organizations to enhance the overall well-being of sports professionals. This initiative is expected to improve job satisfaction, which in turn may foster improvements in both short-term and long-term performance for coaches and athletes alike.

### 4.2. Second Hypothesis

We anticipated that higher levels of perceived stress would be associated with increased burnout among coaches. However, our findings do not support Hypothesis 2, as they reveal a significant inverse relationship between Perceived Distress (β = −0.369; *p* < 0.0001), Perceived Coping (β = −0.139; *p* < 0.05), Perceived Anxiety (β = −0.328; *p* < 0.0001), and Emotional and Physical Exhaustion. Furthermore, no statistically significant relationship was observed between Perceived Distress (β = −0.107; *p* = 0.155), Perceived Coping (β = −0.055; *p* = 0.432), Perceived Anxiety (β = −0.111; *p* = 0.062), and Reduced Sense of Accomplishment, or between Perceived Distress (β = −0.052; *p* = 0.449), Perceived Anxiety (β = −0.104; *p* = 0.110), and Sport Devaluation. A unique positive, though not significant, relationship was found between Perceived Coping and Sport Devaluation (β = 0.102; *p* = 0.121). Interestingly, coaches with higher stress levels are less likely to experience burnout. This finding contradicts previous research that reported a significant positive association between overall perceived stress and burnout subscales in NCAA collegiate coaches, but it aligns with studies conducted among students [77,78]. The discrepancy may be attributed to methodological factors, as the sampling periods varied across different sport seasons. Consequently, coaches in different sports may have perceived and reported stress differently. Future research might consider longitudinal designs to better capture the temporal dynamics between stress and burnout in this specific context.

### 4.3. Third Hypothesis

Only partial support was found for Hypothesis 3. Our research showed that mental toughness buffered the relationship between Perceived Coping and all burnout subfactors. Additionally, mental toughness negatively impacted the relationship between Perceived Distress, Perceived Anxiety, Reduced Sense of Accomplishment, and Sport Devaluation. However, it did not buffer the relationship between Perceived Distress, Perceived Anxiety, and Emotional and Physical Exhaustion. This study extends previous research by demonstrating that mental toughness can protect individuals from stress. This may be because mentally tough individuals tend to view their lives as more controllable, which leads them to feel capable of influencing their circumstances and controlling their destiny [33,44]. Furthermore, mental toughness can act as a protective factor by enhancing individuals’ emotional regulation and coping strategies [79]. 

Our findings are consistent with earlier observations made in both athletic and non-athletic populations, further supporting the notion of mental toughness as a vital coping resource for athletes [43,46,80]. In summary, mental toughness serves as a buffer between stress and burnout; however, the buffering effects of its various subfactors on different dimensions of coaching burnout vary. Consequently, further research is necessary to fully understand the mitigating effects of mental toughness, particularly by analyzing coping strategies, motivation, and success.

### 4.4. Limits and Perspectives

Our research could be among the first examples to focus on coaches by examining the relationship between stress, burnout, and mental toughness. However, this study has several limitations. Firstly, it employs a cross-sectional design, meaning that he observed results do not establish causality or demonstrate a cause-and-effect relationship. Future research could benefit from a longitudinal design to explore the causal relationships between perceived stress, mental toughness, and burnout over time, as well as cross-cultural replication or interventions aimed at enhancing mental toughness.

Secondly, the original data were collected through a questionnaire survey, and reliance on self-reported measurements may influence the results regarding the relationships between the variables. Understanding the systematic biases inherent in self-report measures is therefore an essential element of any methodological critique of survey-based research, particularly when self-reporting is used to assess all variables within a study—an occurrence that is not uncommon in sport psychology.

Thirdly, given that the alpha coefficient for the CBQ is 0.69 and for the SMTQ is 0.64—both falling below the commonly accepted threshold of 0.70—this indicates that the internal consistency of these measures is suboptimal. Despite numerous studies indicating that a Cronbach’s alpha of 0.6 or lower is acceptable [81], this should be acknowledged as a limitation.

Fourthly, many foreign coaches working in Hungary could not complete our questionnaire due to a lack of language proficiency. This language barrier hindered their ability to fully comprehend the questions or accurately articulate their responses, ultimately leading to a decreased response rate within this group and potentially affecting the completeness and representativeness of our data. Despite these limitations, the findings highlight the significance of stress management training for coaches in addressing emotional demands and mitigating the risks of burnout. Practical applications may include workshops on burnout that emphasize self-awareness, emotion regulation, and the process of letting go. Future research should explore the effectiveness of such interventions through longitudinal studies.

## 5. Conclusions

This study represents the inaugural application of Smart PLS to investigate the mediating effect of mental toughness on the relationship between perceived stress and burnout among Hungarian coaches. The current investigation offers a more comprehensive analysis of these factors compared to previous studies conducted within this population, thereby enhancing the understanding of the coach burnout process. As a result, this opens up opportunities for the development of stress management training and workshops for coaches to safeguard their mental health.

## Figures and Tables

**Table 1:** Demographic characteristics.

Variables	N = 333
Age	43.08 ± 12.95
Gender	
Female	108 (32%)
Male	225 (68%)
Level	
First Class and National Team	164 (49%)
Lower Classes	169 (51%)
Coaching experience	
Debutant (1–5 years)	75 (23%)
Average experience (6–10 years)	81 (24%)
Experienced (+10 years)	177 (53%)

**Table 2:** The classification of coaches participating in the study by sport discipline.

Aerobics	5	Jiu-Jitsu	1
American Football	1	Judo	20
Athletics	13	Karate	3
Badminton	1	Orienteering	1
Basketball	25	Pentathlon	2
Beach Volleyball	1	Personal Training	3
Boxing	4	Rhythmic Gymnastics	6
Canoeing	11	Sailing	7
Cycling	3	Speed skating	3
Dance	3	Swimming	12
Fencing	12	Synchronized Swimming	3
Figure Skating	1	Table Tennis	5
Football	36	Tennis	2
Futsal	6	Triathlon	10
Gymnastics	5	Volleyball	24
Handball	95	Water Polo	7
Ice Hockey	1	Wrestling	1

**Table 3:** Descriptive statistics of study variables.

	Mean	SD	Skewness	Kurtosis
Overall Burnout	3.91	0.60	−0.70	0.70
Overall Mental Toughness	44.69	5.19	−0.65	1.24
Overall Stress	21.09	7.54	0.51	0.42

**Table 4:** Comparison of the Hungarian averages of burnout subfactors with international data.

Authors	Country	International Studies Mean	International Studies SD	Present Study Mean	Present Study SD	Report	Cronbach α
	Emotional and Physical Exhaustion						
Harris and Ostrow, 2008 [56]	USA	2.73	0.98	3.98	0.78	greater *p* < 0.001	0.94
Kilo and Hassmén, 2016 [67]	Australia	2.39	0.95				0.93
Lundkvist et al., 2016 [68]	Sweden	2.12	0.05				0.90
Ong and Zhao, 2018 [69]	Singapore	2.35	0.82				0.91
Woodruff, 2022 [70]	United Kingdom	2.23	0.95				0.93
Malinauskas and Malinauskiene, 2023 [29]	Lithuania	2.16	0.62				0.76
Bíró et al., 2025 [57]	Hungary	2.04	1.10				0.89
	Reduced Sense of Accomplishment						
Harris and Ostrow, 2008 [56]	USA	2.73	0.67	3.82	0.64	greater *p* < 0.001	0.81
Kilo and Hassmén, 2016 [67]	Australia	2.02	0.62				0.80
Ong and Zhao, 2018 [69]	Singapore	2.04	0.63				0.82
Woodruff, 2022 [70]	United Kingdom	2.16	0.61				0.77
Malinauskas and Malinauskiene, 2023 [29]	Lithuania	2.64	0.46				0.77
Bíró et al., 2025 [47]	Hungary	2.93	0.98				0.65
	Sport Devaluation						
Harris and Ostrow, 2008 [56]	USA	2.03	0.84	3.84	0.84	greater *p* < 0.001	0.88
Kilo and Hassmén, 2016 [67]	Australia	1.97	0.73				0.84
Ong and Zhao, 2018 [69]	Singapore	1.93	0.69				0.81
Woodruff, 2022 [70]	United Kingdom	1.96	0.77				0.84
Malinauskas and Malinauskiene, 2023 [29]	Lithuania	2.08	0.46				0.71
Bíró et al., 2025 [47]	Hungary	2.20	1.18				0.66

Note. The comparison of Hungarian data to the average values of international research.

**Table 5:** The mediating effect of Mental Toughness between Perceived Stress and Coach Burnout.

	Original Sample (O)	Sample Mean (M)	Standard Deviation (STDEV)	T Statistics (|O/STDEV|)	*p* Values
(TIE) PS → CB	−0.169	−0.175	0.053	3.158	0.002
Direct effect					
PS → CB	−0.382	−0.383	0.075	5.089	0.000
PS → MT	−0.692	−0.699	0.023	29.711	0.000
MT → CB	0.244	0.251	0.077	3.155	0.002
Total Effect	−0.550	−0.558	0.045	12.224	0.000

Note. PS = Perceived Stress, CB = Coach Burnout, and MT = Mental Toughness.

**Table 6:** The mediating effect of Mental Toughness between stress factors and Emotional and Physical Exhaustion.

	Original Sample (O)	Sample Mean (M)	Standard Deviation (STDEV)	T Statistics (|O/STDEV|)	*p* Values
(TIE) PD→ EPE	−0.132	−0.133	0.042	3.101	0.002
PD → Confidence → EPE	0.051	0.051	0.025	1.998	0.046
PD → Constancy → EPE	−0.122	−0.123	0.046	2.617	0.009
PD → Control → EPE	−0.060	−0.061	0.022	2.730	0.006
Direct effect	−0.369	−0.371	0.064	5.772	0.000
Total Effect	−0.501	−0.505	0.051	9.891	0.000
(TIE) PC → EPE	−0.194	−0.199	0.049	3.926	0.000
PC → Confidence → EPE	0.064	0.064	0.035	1.817	0.069
PC → Constancy → EPE	−0.185	−0.189	0.051	3.649	0.000
PC → Control → EPE	−0.073	−0.074	0.020	3.611	0.000
Direct effect	−0.139	−0.137	0.070	1.995	0.046
Total Effect	−0.334	−0.337	0.057	5.867	0.000
(TIE) PA → EPE	−0.131	−0.134	0.040	3.239	0.001
PA → Confidence → EPE	0.048	0.048	0.023	2.137	0.033
PA → Constancy → EPE	−0.121	−0.123	0.043	2.837	0.005
PA → Control → EPE	−0.058	−0.059	0.018	3.204	0.001
Direct effect	−0.328	−0.327	0.062	5.249	0.000
Total Effect	−0.458	−0.462	0.050	9.247	0.000

Note. PD = Perceived Distress, PC = Perceived Coping, PA = Perceived Anxiety, and EPE = Emotional and Physical Exhaustion.

**Table 7:** The mediating effect of Mental Toughness between stress factors and Reduced Sense of Accomplishment.

	Original Sample (O)	Sample Mean (M)	Standard Deviation (STDEV)	T Statistics (|O/STDEV|)	*p* Values
(TIE) PD → RSA	−0.267	−0.273	0.044	6.023	0.000
PD → Confidence → RSA	−0.121	−0.123	0.026	4.609	0.000
PD → Constancy → RSA	−0.075	−0.078	0.039	1.900	0.057
PD → Control → RSA	−0.071	−0.072	0.025	2.879	0.004
Direct effect	−0.107	−0.103	0.075	1.424	0.155
Total Effect	−0.373	−0.376	0.056	6.609	0.000
(TIE) PC → RSA	−0.317	−0.321	0.046	6.923	0.000
PC → Confidence → RSA	−0.154	−0.155	0.033	4.720	0.000
PC → Constancy → RSA	−0.100	−0.104	0.042	2.363	0.018
PC → Control → RSA	−0.062	−0.063	0.019	3.211	0.001
Direct effect	−0.055	−0.054	0.070	0.786	0.432
Total Effect	−0.372	−0.375	0.056	6.692	0.000
(TIE) PA → RSA	−0.240	−0.245	0.038	6.321	0.000
PA → Confidence → RSA	−0.115	−0.117	0.025	4.578	0.000
PA → Constancy → RSA	−0.071	−0.074	0.036	1.961	0.050
PA → Control → RSA	−0.053	−0.054	0.018	2.949	0.003
Direct effect	−0.111	−0.109	0.059	1.866	0.062
Total Effect	−0.350	−0.354	0.051	6.900	0.000

Note. PD = Perceived Distress, PC = Perceived Coping, PA = Perceived Anxiety, and RSA = Reduced Sense of Accomplishment.

**Table 8:** The mediating effect of Mental Toughness between stress factors and Sport Devaluation.

	Original Sample (O)	Sample Mean (M)	Standard Deviation (STDEV)	T Statistics (|O/STDEV|)	*p* Values
(TIE) PD → SD	−0.187	−0.194	0.040	4.642	0.000
PD → Confidence → SD	−0.064	−0.066	0.029	2.196	0.028
PD → Constancy → SD	−0.129	−0.134	0.049	2.644	0.008
PD → Control → SD	0.006	0.005	0.029	0.209	0.834
Direct effect	−0.052	−0.050	0.069	0.758	0.449
Total Effect	−0.240	−0.245	0.054	4.423	0.000
(TIE) PC → SD	−0.279	−0.288	0.046	6.140	0.000
PC → Confidence → SD	−0.096	−0.099	0.038	2.500	0.012
PC → Constancy → SD	−0.178	−0.183	0.053	3.358	0.001
PC → Control → SD	−0.005	−0.006	0.022	0.244	0.807
Direct effect	0.102	0.108	0.066	1.549	0.121
Total Effect	−0.177	−0.181	0.056	3.180	0.001
(TIE) PA → SD	−0.163	−0.169	0.037	4.453	0.000
PA → Confidence → SD	−0.058	−0.060	0.028	2.035	0.042
PA → Constancy → SD	−0.109	−0.113	0.045	2.430	0.015
PA → Control → SD	0.004	0.003	0.019	0.208	0.835
Direct effect	−0.104	−0.103	0.065	1.599	0.110
Total Effect	−0.267	−0.272	0.049	5.471	0.000

Note. PD = Perceived Distress, PC = Perceived Coping, PA = Perceived Anxiety, and SD = Sport Devaluation.

## Data Availability

The data that support the findings of this study are available from the corresponding author, E.B., upon reasonable request. The data are not publicly available due to privacy and ethical restrictions.

## Abbreviations

The following abbreviations are used in this manuscript:

CBQ	Coach Burnout Questionnaire
SMTQ	Sports Mental Toughness Questionnaire
PSS	Perceived Stress Scale
PSS	Perceived Stress
CB	Coach Burnout
MT	Mental Toughness
PD	Perceived Distress
PC	Perceived Coping
PA	Perceived Anxiety
EPE	Emotional and Physical Exhaustion
RSA	Reduced Sense of Accomplishment
SD	Sport Devaluation
